# Human-specific activation of the DUX4-SLC34A2 axis by herpesviruses suppresses antiviral innate immunity

**DOI:** 10.1128/mbio.02554-25

**Published:** 2025-11-10

**Authors:** Ming Gao, Xi Cheng, Chuchu Zhang, Jiali Ma, Xuezhang Tian, Shaowei Wang, Xiaoyu Xie, Yunhong Zhong, Siyuan Wang, Pinghui Feng, Junjie Zhang

**Affiliations:** 1 State Key Laboratory of Oral & Maxillofacial Reconstruction and Regeneration, Key Laboratory of Oral Biomedicine Ministry of Education, Hubei Key Laboratory of Stomatology, State Key Laboratory of Virology, Medical Research Institute, Wuhan University 619779 , Wuhan, China; 2 Frontier Science Center for Immunology and Metabolism, Medical Research Institute, Wuhan University 619779 , Wuhan, China; 3 Hubei Key Laboratory of Tumor Biological Behavior, Hubei Province Cancer Clinical Study Center, Zhongnan Hospital of Wuhan University 89674, Wuhan, China; 4 Department of Colorectal and Anal Surgery, Clinical Center of Intestinal and Colorectal Diseases of Hubei Province, Zhongnan Hospital of Wuhan University 89674, Wuhan, China; 5 Hubei Key Laboratory of Intestinal and Colorectal Diseases, Zhongnan Hospital of Wuhan University 89674, Wuhan, China; 6 Section of Infection and Immunity, Herman Ostrow School of Dentistry, Norris Comprehensive Cancer Center, University of Southern California 5116, Los Angeles, USA; The University of North Carolina at Chapel Hill, Chapel Hill, North Carolina, USA

**Keywords:** herpesvirus, antiviral innate immunity, immune evasion, phosphate

## Abstract

Viral pathogens employ diverse strategies to antagonize host antiviral innate immune defenses. However, the human-specific nature of viral immune evasion mechanisms remains poorly understood. Here, we report that herpesvirus infection selectively activates the embryonic transcription factor DUX4 in human but not murine cells. DUX4 drives the expression of the phosphate transporter SLC34A2, which plays a critical role in suppressing antiviral innate immunity. Mechanistically, SLC34A2 increases intracellular phosphate levels, thereby suppressing the activity of multiple immune and stress-related kinases, including TBK1. Genetic disruption of DUX4 or SLC34A2 restores innate immune activation and enhances interferon responses. Our findings reveal a previously unrecognized, phosphate-mediated immunosuppressive mechanism and define a human-specific transcriptional circuit exploited by herpesviruses to antagonize innate immunity.

**IMPORTANCE:**

Herpesviruses are notorious for their ability to evade host immune responses, yet the mechanisms underlying human-specific immune evasion remain poorly understood. This study identifies a previously unrecognized viral immune evasion strategy by which herpesviruses suppress antiviral immunity in human cells but not murine cells. We demonstrate that herpesvirus infection induces the expression of the embryonic transcription factor DUX4, which subsequently activates its downstream target, SLC34A2, a phosphate transporter. DUX4-SLC34A2 activation reprograms infected cells toward an embryonic-like transcriptional profile, creating an environment conducive to viral replication. Importantly, we show that SLC34A2 increases intracellular phosphate levels, thereby suppressing the activity of multiple immune and stress-related kinases, including TBK1. Our findings reveal a previously unrecognized phosphate-mediated regulation of antiviral immunity, providing insights into viral-host interactions and highlighting therapeutic targets for enhancing antiviral defense.

## INTRODUCTION

Innate immunity represents the first line of defense against invading viral pathogens (1). Germline-encoded pattern recognition receptors (PRRs) are responsible for recognizing pathogen-associated molecular patterns (PAMPs) and damage-associated molecular patterns (DAMPs) associated with virus infection or cellular damage (2–4). The signaling pathways triggered by these PRRs converge on the activation of key innate immune kinases, such as TBK1 and transcription factors, including IRF3 and NF-κB, culminating in the induction of interferons, inflammatory cytokines, and interferon-stimulated genes (ISGs) (5–7). Together, these downstream products function cooperatively to limit viral replication and orchestrate the activation of adaptive immune system (8).

Viruses have co-evolved with the hosts for millions of years and developed sophisticated strategies to evade or subvert host innate defenses (9, 10). While much is known about how viruses target specific components of the innate immune system, the nature of human-specific viral immune evasion mechanisms remains poorly characterized. These virus-host arms races are shaped by long-standing evolutionary virus-host interactions (11, 12), in which viruses adapt to exploit host-specific vulnerabilities. However, such adaptations are ineffective in other species due to critical differences in innate immune regulators or restriction factors. For example, HIV-1 Vpu protein effectively antagonizes the restriction factor Tetherin/BST2 in humans and chimpanzees, but not in rhesus macaques (13, 14). Similarly, influenza A virus NS1 protein targets the ubiquitin E3 ligases TRIM25 and Riplet in a human-specific manner to inhibit RIG-I-mediated antiviral innate immune responses (15). In the cGAS-STING DNA sensing pathway, herpes simplex virus UL37 deamidates human cGAS but not cGAS from most non-human primates, whereas dengue virus viral protease NS2B3 cleaves human STING but fails to do so in non-human primates (16, 17). These studies highlight how viruses directly target key restriction factors or innate signaling molecules in a human-specific manner for immune evasion (9, 10).

Beyond direct targeting innate defense factors, recent studies have revealed that viral pathogens, particularly herpesviruses, can reprogram the infected cells to an unprecedented scale. For example, human cytomegalovirus (HCMV) infection subverts macrophage identity by upregulating the transcription factors ZEB1 and SNAI2, thereby increasing their stemness and facilitating viral dissemination (18). In addition, onco-herpesvirus Kaposi’s sarcoma-associated herpesvirus (KSHV) infection induces transitions between endothelial and mesenchymal states, depending on the specific cell types initially infected. The transition process is believed to play a key role in KSHV-associated tumorigenesis (19–21). Such reprogramming processes typically rely on key transcription factors, which play pivotal roles in reshaping cell identity (22, 23). However, whether viruses exploit such reprogramming strategies to suppress antiviral innate immunity in a human-specific manner remains unclear.

In this study, we show that herpesvirus infection induces the DUX4-SLC34A2 axis in human but not murine cells. DUX4 drives the expression of the phosphate transporter SLC34A2, which plays a critical role in restricting antiviral innate immunity. SLC34A2 elevates intracellular phosphate levels, thereby suppressing the activity of multiple immune and stress-related kinases, including TBK1. Collectively, this study reveals a human-specific immune evasion strategy employed by herpesviruses and uncovers a previously unrecognized role of SLC34A2 in counteracting innate immunity.

## RESULTS

### HSV-1 infection induces DUX4, which is critical for viral replication

**Fig 1:**
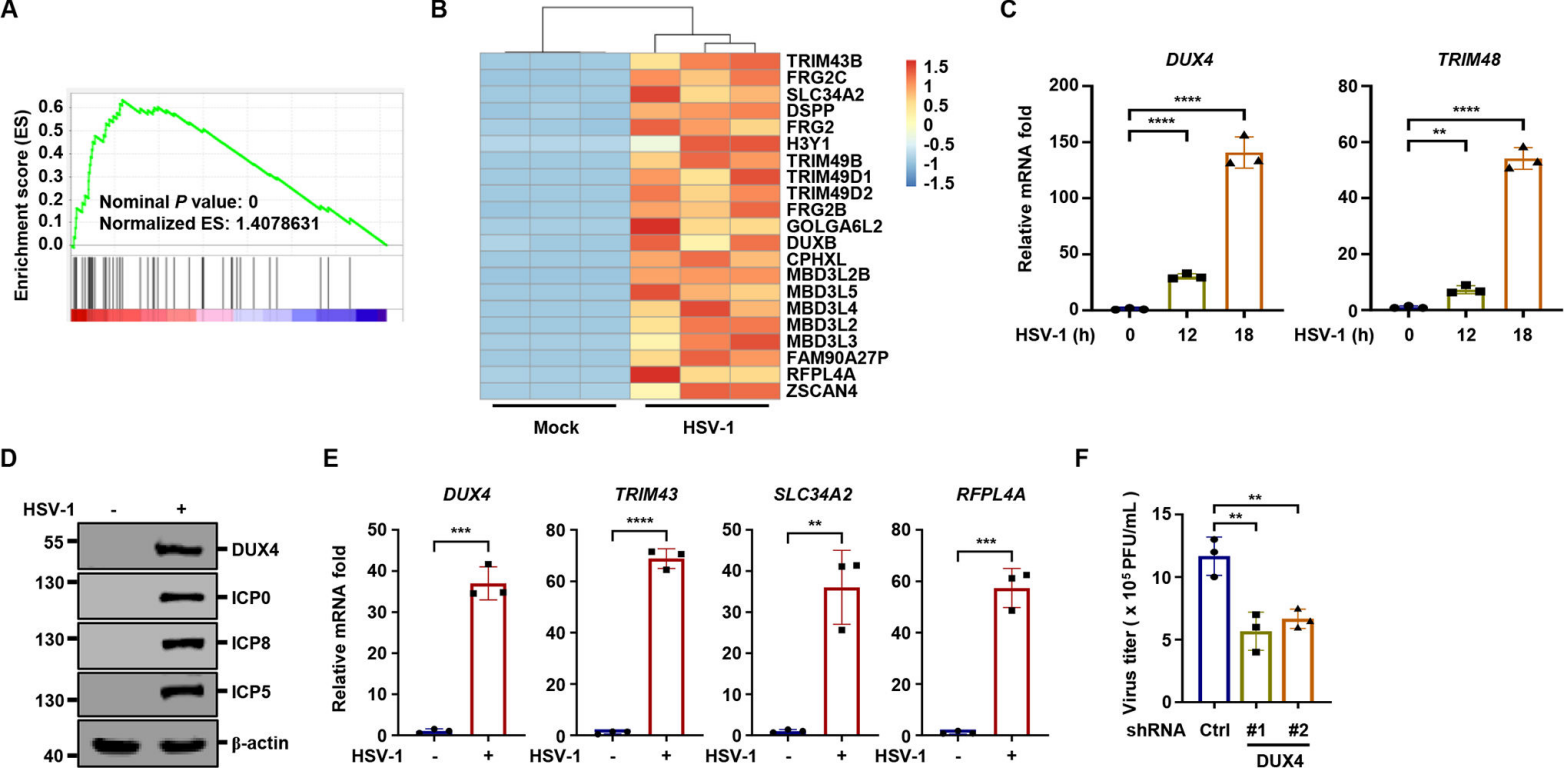
HSV-1 infection induces DUX4 (**A and B**) THP-1 cells were mock-infected or infected with HSV-1 (MOI = 1) for 12 h, followed by RNA-seq analysis. GSEA analysis showed that the DUX4 signaling pathway was highly enriched (**A**). A heatmap showed that a series of DUX4 downstream genes were induced by HSV-1 (**B**). (**C**) THP-1 cells were infected with HSV-1 (MOI = 1), and the expression of *DUX4* and *TRIM48* was quantified at 12 and 18 h post-infection. (**D**) THP-1 cells were infected with HSV-1 (MOI = 1), and whole cell lysates (WCLs) were analyzed by immunoblotting at 12 h post-infection. (**E**) HFF cells were infected with HSV-1 (MOI = 1), and the expression of the indicated genes was quantified at 12 h post-infection. (**F**) HFF cells transduced with control shRNA (Ctrl) or shRNA targeting *DUX4* were infected with HSV-1 (MOI = 0.05). Viral titers in the supernatants were quantified at 36 h post-infection.

To investigate the transcriptional changes induced by HSV-1, we infected the THP-1 monocyte cell line with HSV-1 and performed RNA sequencing. Gene set enrichment analysis (GSEA) indicated that DUX4 signaling was potently activated by HSV-1 (Fig. 1A), consistent with previous reports (24). Heatmap analysis also revealed that a subset of DUX4 downstream genes was significantly induced by HSV-1 (Fig. 1B). Next, we validated that both *DUX4* and its downstream gene *TRIM48* were markedly upregulated following HSV-1 infection (Fig. 1C). Moreover, ZSCAN4 promoter reporter activity, which reflects DUX4 transcriptional activity (25), was greatly enhanced following HSV-1 infection (Fig. S1A). Immunoblotting analysis further confirmed the induction of DUX4 protein by HSV-1 (Fig. 1D). Interestingly, previous studies have shown that herpesvirus infection induces TRIM43 in a DUX4-dependent manner and that TRIM43 restricts herpesvirus replication (24). We confirmed that knockdown of *DUX4* in primary human foreskin fibroblasts (HFFs) significantly reduced *TRIM43* induction by HSV-1 (Fig. S1B and C). In addition, knockdown of *TRIM43* in HFF cells significantly promoted HSV-1 gene transcription and progeny virion production (Fig. S1D through G), consistent with previous studies (24). These results confirm that TRIM43, induced by DUX4, restricts HSV-1 replication. Furthermore, HSV-1 infection robustly induced the expression of *DUX4* and its downstream genes in HFFs (Fig. 1E), and knockdown of DUX4 significantly impaired HSV-1 replication (Fig. 1F). Collectively, these data indicate that HSV-1 infection induces DUX4 expression, which is critical for viral replication.

### Human-specific induction of DUX4 by herpesviruses

**Fig 2:**
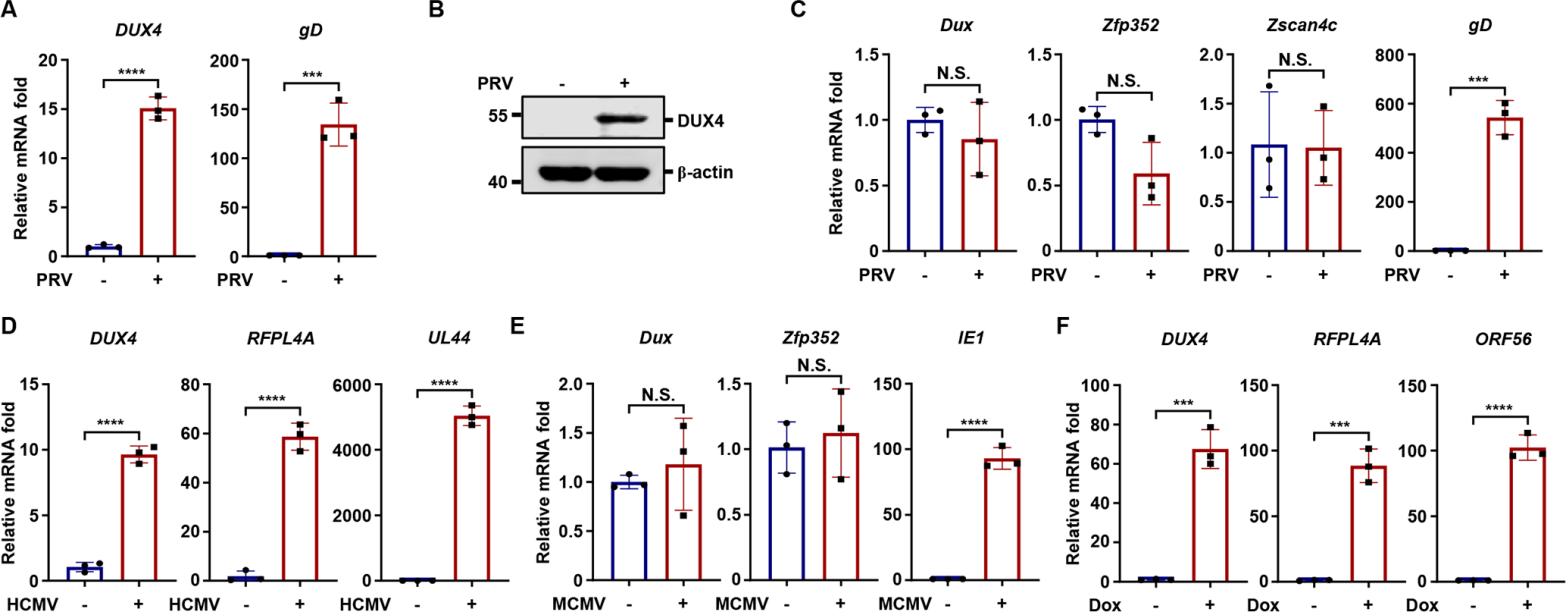
Species-specific induction of DUX4 by herpesviruses (**A and B**) U2OS cells were infected with PRV (MOI = 1). The expression of the indicated genes was quantified at 24 h post-infection (**A**), and WCLs were analyzed by immunoblotting at the indicated time points post-infection (**B**). (**C**) MLF cells were infected with PRV (MOI = 1), and the expression of the indicated genes was quantified at 24 h post-infection. (**D**) HFF cells were infected with HCMV (MOI = 0.5), and the expression of the indicated genes was quantified at 5 days post-infection. (**E**) MLF cells were infected with MCMV (MOI = 1), and the expression of the indicated genes was quantified at 3 days post-infection. (**F**) BCBL-1-Tet-RTA cells were treated with Dox (1 µg/mL) and sodium butyrate (0.2 mM). The expression of the indicated genes was quantified at 24 h post-induction.

Interestingly, we observed that HSV-1 infection of primary murine lung fibroblasts (MLFs) did not induce *Dux*, the murine homolog of human *DUX4,* or its downstream genes (Fig. S2A), suggesting human-specific induction of DUX4 by HSV-1. Therefore, we investigated whether DUX4 can be induced by other viruses and whether the induction is conserved across human and mouse cells. Notably, pseudorabies virus (PRV) infection induced DUX4 expression in human osteosarcoma U2OS cells (Fig. 2A and B). In contrast, PRV failed to induce *Dux* in murine MLFs despite efficient viral replication (Fig. 2C). Similarly, HCMV infection robustly upregulated DUX4 and its downstream genes in human HFFs (Fig. 2D). Given the strict host specificity of HCMV, we examined whether murine cytomegalovirus (MCMV) could induce *Dux* in murine cells. Notably, MCMV infection failed to activate *Dux* in MLF cells (Fig. 2E). Next, we found that lytic reactivation of KSHV strongly induced *DUX4* and its downstream gene *RFPL4A*, consistent with previous studies (24) (Fig. 2F). In contrast, infection with murine herpesvirus MHV68 did not induce *Dux* or its downstream genes in murine MLFs (Fig. S2B). Interestingly, MCMV infection of A549 cells and MHV68 infection of HEK293T cells induced the expression of DUX4 and its downstream gene TRIM48 (Fig. S2C and D), indicating that murine herpesviruses can activate DUX4 in human cells.

To further assess the specificity of DUX4 induction by herpesviruses, we tested additional DNA and RNA viruses. Human adenovirus (HAdV) and vaccinia virus (VACV) infections could not induce *DUX4* expression (Fig. S2E and F). Additionally, vesicular stomatitis virus (VSV) and Sendai virus (SeV), two RNA viruses, did not activate *DUX4* (Fig. S2G). These data collectively indicate that herpesvirus infection specifically induces DUX4 in human cells but not in murine cells.

### DUX4 suppresses antiviral innate immunity dependent on its transcriptional activity

**Fig 3:**
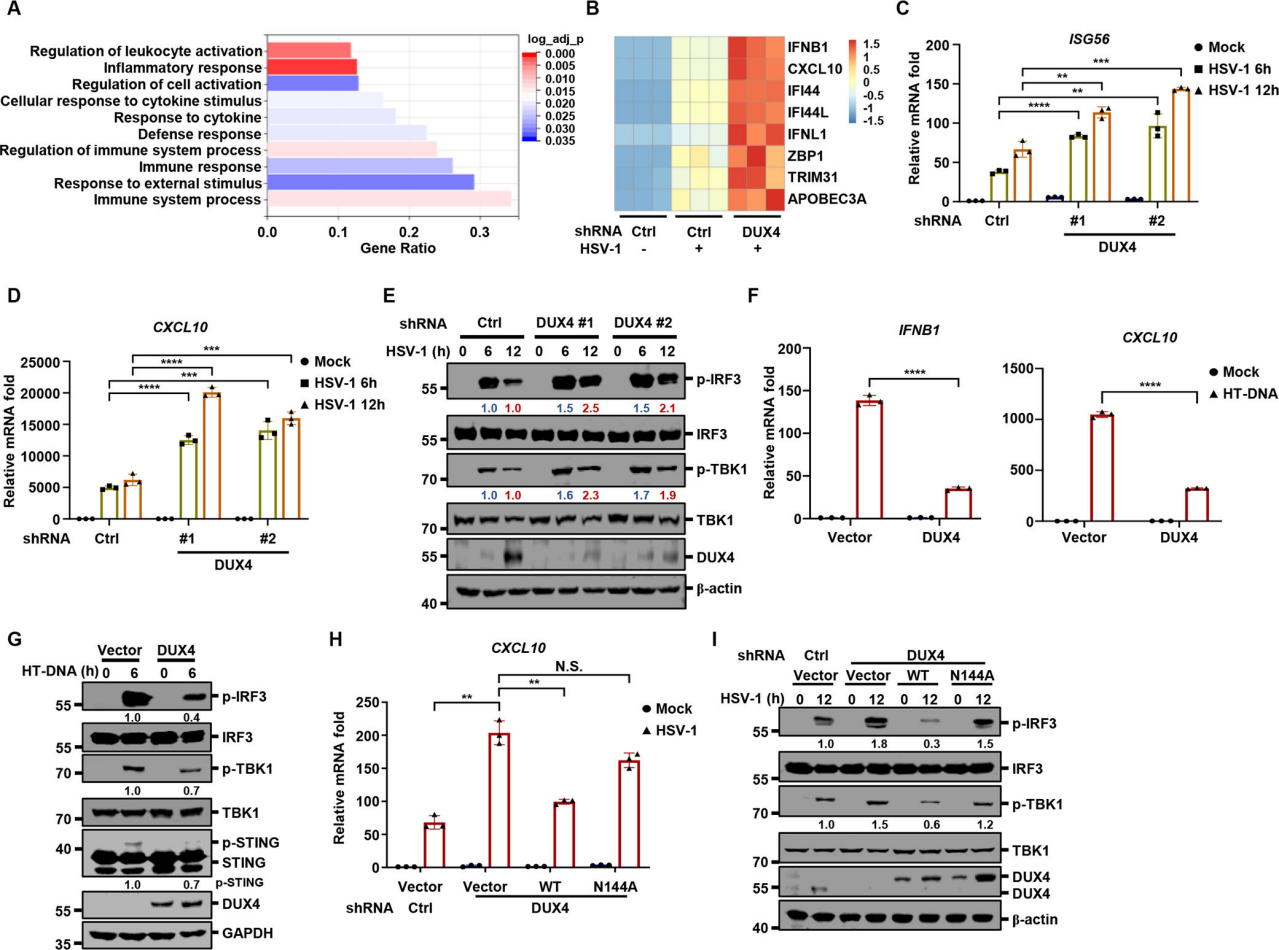
DUX4 suppresses antiviral innate immunity dependent on its transcriptional activity (**A and B**) THP-1 cells transduced with control shRNA (Ctrl) or shRNA targeting *DUX4* were mock-infected or infected with HSV-1 (MOI = 1) for 12 h. RNA-seq was performed, and Gene Ontology (GO) analysis showed the enrichment of biological processes related to immune activation and inflammation (**A**). A heatmap showed the expression of innate immune genes (**B**). (**C through E**) THP-1 control or *DUX4* knockdown stable cells were infected with HSV-1 (MOI = 5). The expression of *ISG56* and *CXCL10* was quantified (**C and D**), and WCLs were analyzed by immunoblotting (**E**) at the indicated time points post-infection. (**F and G**) THP-1 cells stably expressing doxycycline-inducible vector control or *DUX4* were treated with doxycycline (500 ng/mL) for 24 h, and then transfected with HT-DNA (1 µg/mL). The expression of *IFNB1* and *CXCL10* was quantified at 6 h post-transfection (**F**), and WCLs were analyzed by immunoblotting at the indicated time points post-transfection (**G**). (**H and I**) THP-1 control or *DUX4* knockdown stable cells were transduced with doxycycline-inducible vector control, DUX4-WT, or the N144A mutant. The stable cell lines were induced with doxycycline (500 ng/mL) for 24 h, followed by infection with HSV-1 (MOI = 5). The expression of *CXCL10* was quantified at 12 h post-infection (**H**), and WCLs were analyzed by immunoblotting at the indicated time points post-infection (**I**).

Gene Ontology (GO) analysis revealed that immune activation and inflammatory responses were among the top upregulated processes upon DUX4 depletion during HSV-1 infection (Fig. 3A). Moreover, heatmap analysis also indicated that DUX4 depletion further enhanced the expression of *IFNB1* and a battery of ISGs (such as *CXCL10* and *ZBP1*) in HSV-1-infected cells (Fig. 3B), suggesting that DUX4 induced by HSV-1 suppresses antiviral innate immunity. Next, we confirmed the RNA sequencing results by showing that knockdown of *DUX4* led to higher induction of ISGs, such as *ISG56* and *CXCL10* by HSV-1 (Fig. 3C and D). Consistently, HSV-1-induced phosphorylation of TBK1 and IRF3 was enhanced in DUX4-depleted cells compared with control cells, coinciding with DUX4 induction (Fig. 3E). Conversely, inducible expression of DUX4 potently suppressed intracellular herring testis (HT)-DNA-induced *IFNB1* and *CXCL10* (Fig. 3F). Immunoblotting analyses further showed that DUX4 inducible expression decreased the phosphorylation of STING, TBK1, and IRF3 induced by intracellular DNA (Fig. 3G). Similarly, HSV-1-induced innate immune responses were also suppressed by inducible expression of DUX4 (Fig. S3A and B). Interestingly, DUX4 overexpression dose-dependently inhibited both cGAS-STING- and TBK1-activated IFNβ promoter-driven reporter activities (Fig. S3C). Additionally, DUX4-inducible expression hampered the nuclear translocation of IRF3 induced by intracellular DNA (Fig. S3D). These results suggest that DUX4 intervenes downstream of cGAS and STING, likely at the level of TBK1 and/or IRF3, to antagonize antiviral innate immune responses. Notably, inducible expression of murine Dux in murine fibroblasts also significantly inhibited antiviral innate immune responses triggered by HSV-1 and VSV (Fig. S3E through G). Together with our prior findings that herpesviruses induce DUX4 specifically in human cells, these results suggest that the inability to induce Dux in murine cells during herpesvirus infection is a major reason for the absence of this immune evasion pathway in mice.

DUX4 is a key transcription factor involved in zygotic genome activation during embryonic development and is typically silenced epigenetically in somatic cells (25–28). Misexpression of DUX4 is particularly toxic to muscle tissue and is etiologically linked to facioscapulohumeral muscular dystrophy (FSHD) (29). Moreover, DUX4 de-repression in cancer cells results in the suppression of antigen presentation and promotes immune evasion (30). Building on previous studies and our own observations, we sought to determine whether the transcriptional activity of DUX4 is required to suppress antiviral innate immunity. To identify a transcription activity-deficient mutant of DUX4, we introduced mutations into the residues of DUX4 potentially involved in DNA binding, generating a series of DUX4 mutants (31, 32). ZSCAN4 promoter reporter assays revealed that the N144A mutation of DUX4 completely abolished its transcriptional activity (Fig. S3H). As a control, N69A did not influence the transcriptional activation of DUX4 (Fig. S3H). Subsequently, we reconstituted DUX4-depleted cells with wild-type (WT) DUX4 or the N144A mutant and found that re-expression of WT DUX4, but not N144A, could reverse the enhanced induction of *CXCL10* by HSV-1 in DUX4-depleted cells (Fig. 3H). Moreover, reconstitution of WT DUX4, but not N144A, repressed HSV-1-induced phosphorylation of TBK1 and IRF3 in DUX4 knockdown cells (Fig. 3I). Consistently, the inducible expression of WT DUX4, but not the N144A mutant, potently suppressed *IFNB1* transcription and phosphorylation of STING, TBK1, and IRF3 induced by HT-DNA (Fig. S3I and J). Together, these data indicate that DUX4 induced by HSV-1 suppresses antiviral innate immunity dependent on its transcriptional activity.

### SLC34A2 induced by DUX4 is required to suppress antiviral innate immunity

**Fig 4:**
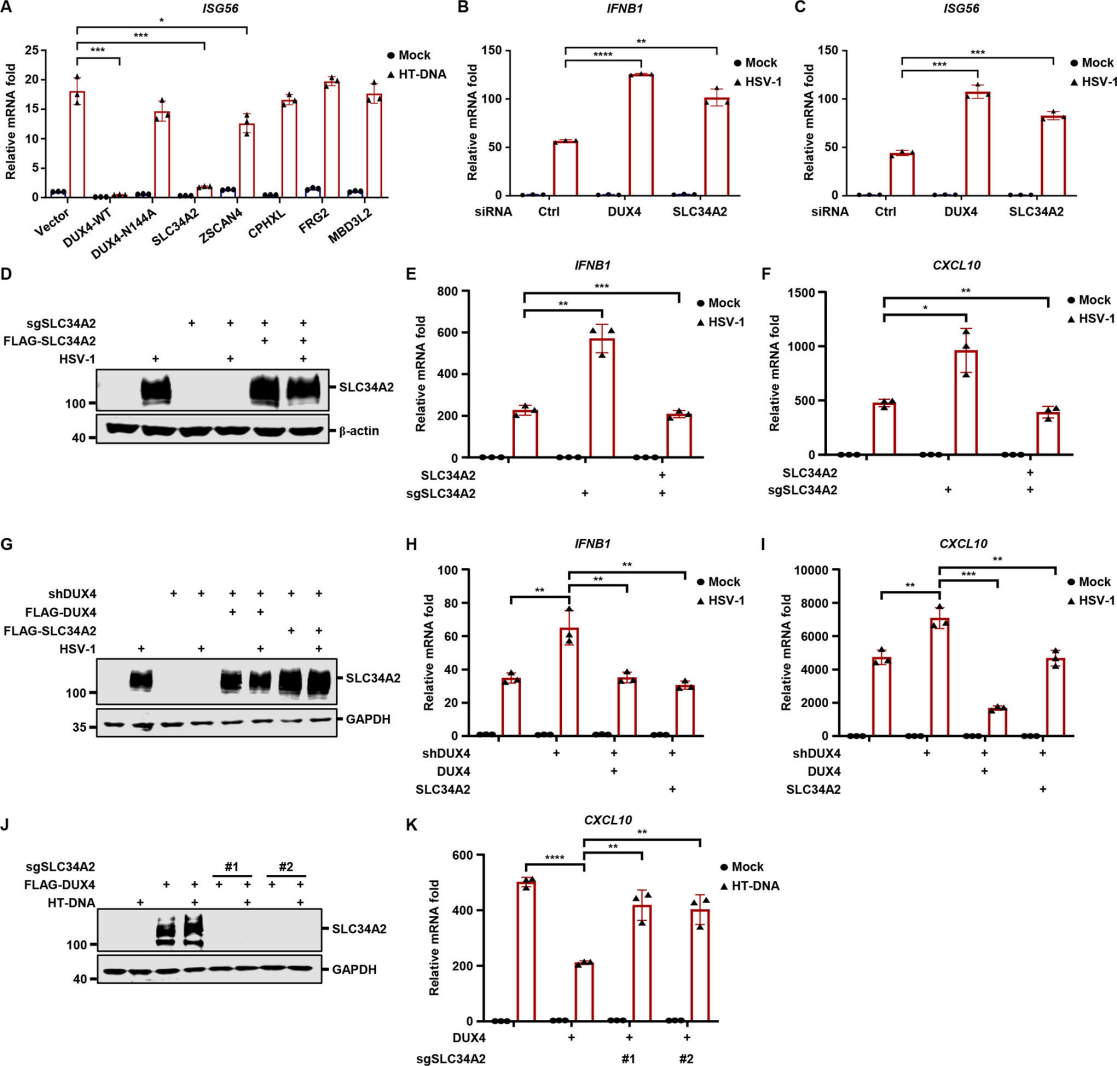
SLC34A2 induced by DUX4 is required to suppress antiviral innate immunity (**A**) THP-1 cells stably expressing doxycycline-inducible vector control or the indicated genes were treated with doxycycline (500 ng/mL) for 24 h, and then transfected with HT-DNA (1 µg/mL). The expression of *ISG56* was analyzed by RT-qPCR at 6 h post-transfection. (**B and C**) Primary human monocytes transfected with control siRNA or siRNA targeting *DUX4* or *SLC34A2* were infected with HSV-1 (MOI = 1) at 16 h post-transfection. The expression of the indicated genes was quantified at 12 h post-infection. (**D through F**) THP-1 *SLC34A2* knockout cells were transduced with doxycycline-inducible vector control or *SLC34A2*. The stable cell lines were induced with doxycycline for 24 h, and then infected with HSV-1 (MOI = 5). WCLs were analyzed by immunoblotting (**D**), and the expression of *IFNB1* and *CXCL10* was quantified (**E and F**) at 12 h post-infection. (**G through I**) THP-1 control or *DUX4* knockdown stable cells were transduced with doxycycline-inducible *DUX4* or *SLC34A2*. The stable cell lines were induced with doxycycline for 24 h, and then infected with HSV-1 (MOI = 5). WCLs were analyzed by immunoblotting (**G**), and the expression of *IFNB1* and *CXCL10* was quantified (**H and I**) at 12 h post-infection. (**J and K**) THP-1 cells stably expressing doxycycline-inducible vector control or *DUX4* were transduced with control sgRNA or sgRNA targeting *SLC34A2*. The stable cells were induced with doxycycline for 24 h, and then transfected with HT-DNA (1 µg/mL). WCLs were analyzed by immunoblotting (**J**), and the expression of *CXCL10* was quantified (**K**) at 6 h post-transfection.

To delineate the downstream genes of DUX4 involved in immune regulation, we conducted a focused sgRNA knockout screen targeting DUX4 downstream genes. Interestingly, knockout of *FRG2*, *CPHXL*, *SLC34A2*, *MBD3L2-5*, and *ZSCAN4* resulted in higher HSV-1-induced *CXCL10* expression (Fig. S4A). Among these, only inducible expression of *SLC34A2* consistently suppressed HT-DNA-induced expression of *IFNB1* and *ISG56* and inhibited the phosphorylation of TBK1 and IRF3, as potently as DUX4 (Fig. 4A; Fig. S4B and C).

We next assessed whether HSV-1 induces SLC34A2 expression in other cell types. HSV-1 infection also triggered the expression of DUX4 and SLC34A2 in SH-SY5Y neuroblastoma cells, a neuron infection model frequently used to study HSV-1 infection (Fig. S4D and E) (33). Additionally, HSV-1 infection of human primary monocytes robustly induced the transcription of *DUX4* and *SLC34A2* (Fig. S4F through H), and knockdown of *DUX4* or *SLC34A2* significantly increased HSV-1-induced innate immune responses in primary monocytes (Fig. 4B and C).

To further validate the role of SLC34A2 in suppressing antiviral innate immunity, we found that re-expression of SLC34A2 reversed the heightened innate immune responses in SLC34A2-depleted or DUX4-depleted cells during HSV-1 infection (Fig. 4D through I). Furthermore, DUX4-inducible expression potently suppressed HT-DNA-induced *CXCL10* transcription, which was completely negated by knockout of *SLC34A2* (Fig. 4J and K). Together, these data indicate that SLC34A2 induced by DUX4 is required to suppress antiviral innate immunity.

### SLC34A2 modulates intracellular inorganic phosphate levels to restrict antiviral innate immunity

**Fig 5:**
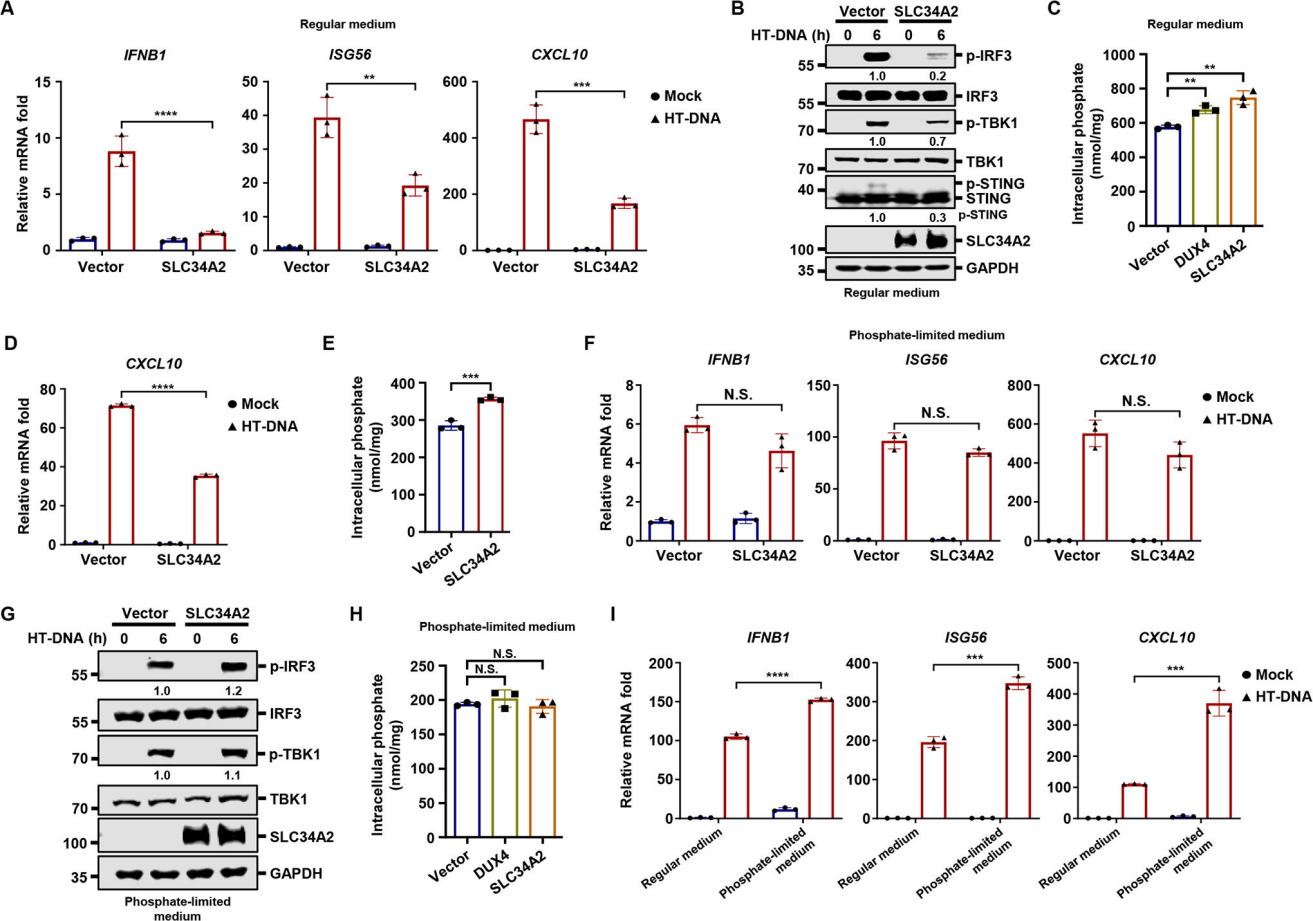
SLC34A2 modulates intracellular inorganic phosphate levels to restrict antiviral innate immunity (**A and B**) THP-1 stably expressing doxycycline-inducible vector control or *SLC34A2* were induced with doxycycline (500 ng/mL) for 24 h under high phosphate culture condition, and then were transfected with HT-DNA (1 µg/mL). The expression of the indicated genes was quantified by RT-qPCR (**A**), and WCLs were analyzed by immunoblotting at 6 h post-transfection (**B**). (**C**) THP-1 stably expressing doxycycline-inducible vector control, *DUX4*, or *SLC34A2* was induced with doxycycline for 24 h under high phosphate culture condition, followed by quantification of intracellular phosphate concentration. (**D**) THP-1 cells stably expressing doxycycline-inducible vector control or *SLC34A2* were induced with doxycycline for 24 h under near-physiological phosphate concentration (10 mg/dL), and then were transfected with HT-DNA (1 µg/mL). The expression of *CXCL10* was quantified by RT-qPCR at 6 h post-transfection. (**E**) THP-1 cells stably expressing doxycycline-inducible vector control or *SLC34A2* were induced with doxycycline for 24 h under near-physiological phosphate concentration (10 mg/dL), followed by quantification of intracellular phosphate concentration. (**F and G**) THP-1 stably expressing doxycycline-inducible vector control or *SLC34A2* were induced with doxycycline (500 ng/mL) for 24 h under phosphate-limited culture condition and then were transfected with HT-DNA (1 µg/mL). The expression of the indicated genes was quantified by RT-qPCR (**F**), and WCLs were analyzed by immunoblotting at 6 h post-transfection (**G**). (**H**) THP-1 stably expressing doxycycline-inducible vector control, *DUX4*, or *SLC34A2* were induced with doxycycline for 24 h under phosphate-limited culture condition, followed by quantification of intracellular phosphate concentration. (**I**) THP-1 cells cultured under high phosphate or phosphate-limited conditions were transfected with HT-DNA (1 µg/mL). The expression of the indicated genes was quantified by RT-qPCR at 6 h post-transfection.

SLC34A2 encodes a sodium-dependent phosphate transporter, and its mutations cause pulmonary alveolar microlithiasis, a rare disease characterized by the deposition of calcium phosphate microliths in the lungs (34, 35). However, whether SLC34A2 is involved in regulating antiviral innate immunity remains unexplored. Interestingly, the inducible expression of SLC34A2 potently suppressed HT-DNA-induced innate immune responses, as evidenced by the reduced transcription of *IFNB1*, *ISG56,* and *CXCL10* (Fig. 5A), and decreased phosphorylation of TBK1 and IRF3 (Fig. 5B). Consistent with its function as a phosphate transporter, both the expression of DUX4 and SLC34A2 significantly increased intracellular inorganic phosphate levels (Fig. 5C). Of note, intracellular phosphate concentrations are tightly regulated through phosphate import via multiple transports, export via XPR1, and buffering by polyphosphate (polyP) (36–38). Therefore, a ~25% increase in intracellular phosphate following DUX4 or SLC34A2 expression is both statistically significant and biologically meaningful for innate immune suppression (Fig. 5F through H).

Since the availability of phosphate in tissue culture medium far exceeds physiological levels, we adapted cell growth to culture medium with near-physiological phosphate concentration (from 50 to 10 mg/dL) (39). Even under these conditions, SLC34A2-inducible expression still significantly repressed HT-DNA-induced antiviral innate immune responses and increased intracellular phosphate levels (Fig. 5D and E), indicating that the suppression of antiviral innate immunity by SLC34A2 is not due to the artifact of high concentrations of extracellular phosphate. Remarkably, when we adapted SLC34A2-expressing cells to phosphate-limited cell culture medium, SLC34A2 failed to suppress HT-DNA-induced innate immune responses (Fig. 5F and G), and intracellular phosphate levels were no longer elevated by DUX4 or SLC34A2 expression (Fig. 5H). Moreover, inducible expression of SLC34A2 also effectively suppressed innate immune responses induced by the dsRNA viral mimic poly I:C (Fig. S5C), in a phosphate-dependent manner (Fig. S5D through F). Furthermore, by culturing cells in either standard or phosphate-limited medium to achieve distinct intracellular phosphate levels (Fig. S5G), we found that low intracellular phosphate levels consistently correlated with higher innate immune activation in response to HT-DNA (Fig. 5I). These findings support the conclusion that elevated intracellular phosphate suppresses antiviral innate immunity.

Collectively, these data indicate that SLC34A2 elevates intracellular phosphate levels to restrict antiviral innate immunity.

### Targeted inhibition of SLC34A2 enhances antiviral innate immune responses

**Fig 6:**
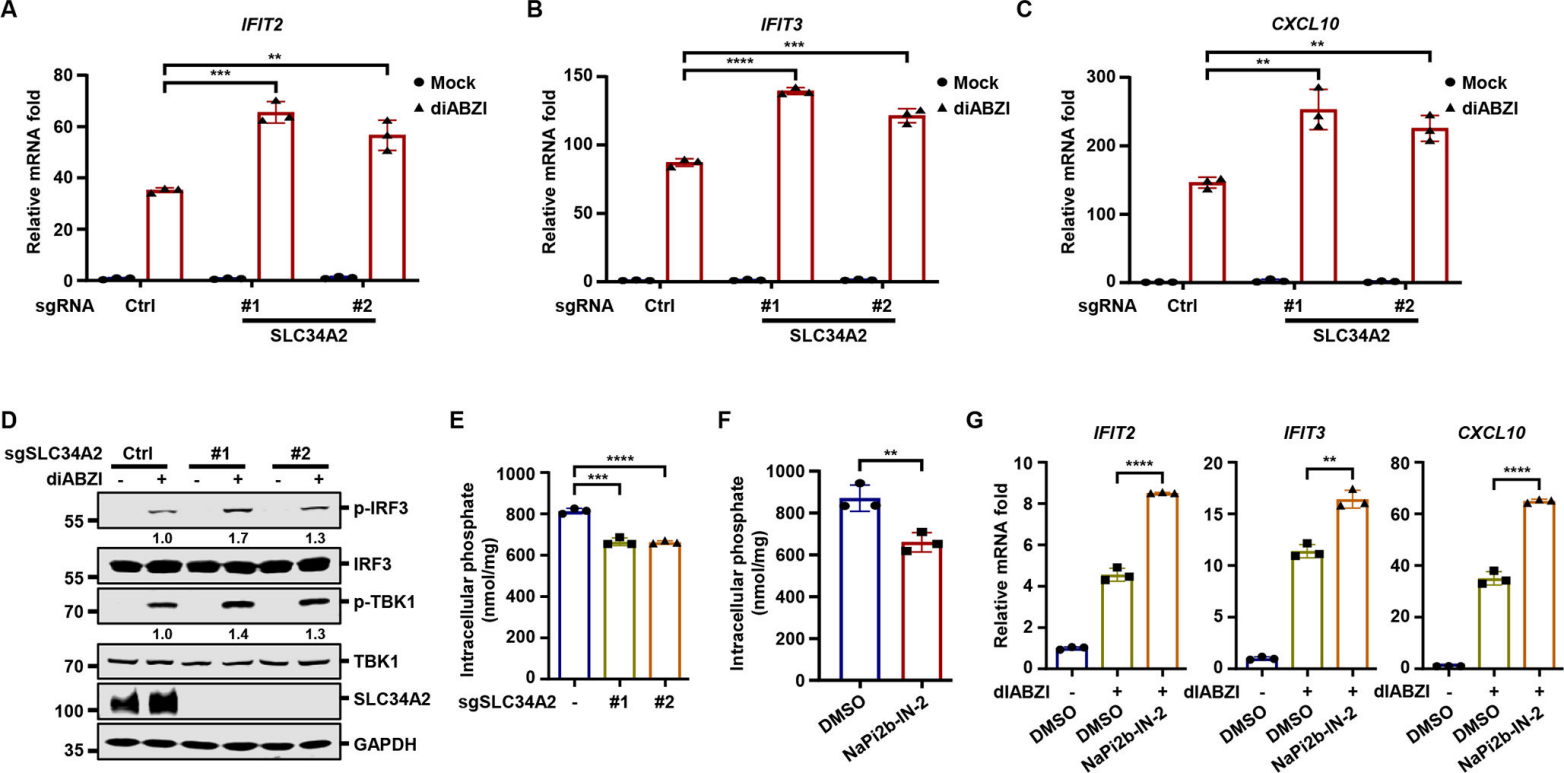
Targeted inhibition of SLC34A2 enhances antiviral innate immune responses (**A through D**) OVCAR-3 cells transduced with control sgRNA or sgRNA targeting *SLC34A2* were stimulated with diABZI (2.5 µM). The expression of the indicated genes was quantified by RT-qPCR (**A through C**), and WCLs were analyzed by immunoblotting at 6 h post-stimulation (**D**). (**E**) Quantification of intracellular phosphate of OVCAR-3 cells transduced with control sgRNA or sgRNA targeting *SLC34A2*. (**F and G**) OVCAR-3 cells were treated with NaPi2b-IN-2 (100 nM) for 40 h, followed by quantification of intracellular phosphate levels (**F**). The treated cells were stimulated with diABZI (2.5 µM), and the expression of the indicated genes was quantified by RT-qPCR at 6 h post-stimulation (**G**).

Next, to determine whether SLC34A2 itself is able to suppress antiviral innate immunity, we turned to an ovarian cancer cell line, OVCAR-3, which endogenously expresses high levels of SLC34A2(40). Indeed, knockout of SLC34A2 in OVCAR-3 increased the expression of *IFIT2*, *IFIT3*, and *CXCL10* induced by the STING agonist diABZI (Fig. 6A through C) (41), as well as the phosphorylation of TBK1 and IRF3 (Fig. 6D). Consistently, knockout of SLC34A2 significantly reduced intracellular phosphate levels in OVCAR-3 cells (Fig. 6E). Notably, when SLC34A2-deficient cells were adapted to phosphate-limited medium, SLC34A2 deficiency no longer enhanced diABZI-induced innate immune responses (Fig. S6A and B), and intracellular phosphate levels were unaffected by SLC34A2 deletion (Fig. S6C). Furthermore, treatment with NaPi2b-IN-2, a specific SLC34A2 inhibitor (42), reduced intracellular phosphate levels (Fig. 6F), and augmented diABZI-induced innate immune responses (Fig. 6G). Moreover, the inducible expression of DUX4 or SLC34A2 did not affect HT-DNA-induced cGAMP production or STING signalosome formation (Fig. S6D and E), ruling out the possibility that DUX4 or SLC34A2 restrict cGAS enzymatic activity or STING adaptor function (43–45). Notably, SLC34A2 expression not only significantly inhibited phosphorylation of TBK1, but also suppressed the phosphorylation of multiple immune and stress-related kinases, including IKKε, JNK, and p38 (Fig. S6F). Given the central role of TBK1 in antiviral innate immunity, we performed *in vitro* kinase assays to evaluate TBK1 kinase activity. Notably, supplementation with phosphate inhibited TBK1 kinase activity when the phosphate concentration exceeded 1 mM, which falls within the range of physiologically relevant intracellular phosphate concentrations (Fig. S6G), suggesting that elevated intracellular phosphate may directly suppress TBK1 activity. Together, these data indicate that genetic or pharmacological inhibition of SLC34A2 reduces intracellular phosphate levels and enhances antiviral innate immune responses.

## DISCUSSION

Viruses, as obligate intracellular pathogens, have engaged in the tug-of-war with the host for hundreds of millions of years. Mammalian cells rely on the innate immune system to detect virus infection and mount antiviral responses, while viruses continuously evolve to evade or subvert antiviral innate defenses (1, 9). Extensive studies have revealed how viruses counteract antiviral innate immunity by directly targeting key innate immune factors. For example, HSV-1 ICP27 interferes with the STING signalsome to inhibit type I IFN expression (46), and VP1-2 deubiquitinates STING to block downstream signaling (47). However, our understanding of human-specific mechanisms of viral immune evasion remains limited. Deciphering these mechanisms not only sheds light on the evolutionary arms race between viruses and their hosts, but also unveils the molecular determinants governing viral host range and interspecies differences in innate immune responses.

In this study, we report human-specific induction of the DUX4-SLC34A2 axis by herpesviruses in human but not murine cells. Consistent with our findings, previous studies have shown that HSV-1 infection induces DUX4 (24, 48). A recent study reports that both human DUX4 and mouse Dux interact with STAT1 and inhibit interferon-stimulated gene (ISG) induction (49). Together with our study, these findings indicate that DUX4 potently suppresses innate immune responses by both repressing the production of interferons and the downstream ISG induction, further highlighting its immunosuppressive role. Moreover, DUX4 has been shown to transcriptionally activate TRIM43, which restricts HSV-1 replication (24). In contrast, here, we show that SLC34A2, another transcriptional target of DUX4, functions to suppress antiviral innate immunity, thereby facilitating viral replication. Therefore, these findings indicate that the downstream effectors of DUX4 can play distinct roles in herpesvirus infection. Notably, our study uncovers a previously unrecognized role of DUX4-SLC34A2 in antagonizing antiviral innate immunity. The mechanism by which herpesviruses induce DUX4 remains unclear, and the human-specific nature of this induction warrants further investigation. Mouse Dux, while sharing similar structural domains with human DUX4, exhibits distinct epigenetic regulatory mechanisms and functions compared with its human counterpart (28, 50). Importantly, forced Dux expression in murine fibroblasts significantly inhibits antiviral innate immune responses, strongly suggesting that the inability of herpesviruses to induce Dux expression in murine cells is a major reason for the absence of this immune evasion pathway in mice. It is plausible that herpesvirus-triggered epigenetic changes are able to induce DUX4 in human cells but fail to activate murine Dux. However, this hypothesis requires further experimental validation.

Our findings also uncover a previously unappreciated immunomodulatory role for SLC34A2, a phosphate transporter. Traditionally, SLC34A2 has been widely recognized as a cancer cell marker in ovarian and lung cancers (51). Preclinical development of an anti-NaPi2b (SLC34A2) antibody-drug conjugate as a therapeutic for non-small cell lung and ovarian cancers has progressed to the clinical stage (40). However, other than its role as a cancer cell marker, the potential contribution of SLC34A2 overexpression to the development of ovarian and lung cancer remains largely unexplored. We provide the first evidence suggesting that SLC34A2 restricts innate immunity, thereby likely suppressing cancer cell immunogenicity and promoting cancer progression (52). Our findings indicate that SLC34A2 is more than just a cancer cell marker but represents a prominent therapeutic target. SLC34A2 inhibition, with blocking antibodies or inhibitors, may hold promise for enhancing antitumor immunity. Moreover, the mechanism by which SLC34A2 suppresses antiviral innate immunity is unique. We have provided compelling evidence that SLC34A2 increases intracellular phosphate levels to antagonize antiviral innate immunity. Elevated intracellular phosphate inhibits multiple immune and stress-related kinases, including TBK1, thereby damping innate immune responses. Therefore, intracellular phosphate homeostasis may function as a previously unrecognized metabolic checkpoint in innate immune regulation. These findings also raise several new questions. For example, the precise mechanism by which elevated intracellular phosphate inhibits innate immunity remains to be elucidated. Moreover, it remains to be explored whether other pumps are involved in the regulation of innate immunity.

In summary, our study reveals that herpesviruses selectively induce the DUX4-SLC34A2 axis in human but not murine cells. The induction of DUX4 establishes an embryonic-like transcriptional program in infected cells, and SLC34A2 increases intracellular phosphate levels, thereby inhibiting the activity of multiple immune and stress-related kinases, including TBK1. Genetic disruption or pharmacological inhibition of SLC34A2 restores innate immune activation and enhances interferon responses. Overall, this study unveils a human-specific immune evasion strategy employed by herpesviruses and uncovers a previously unrecognized role of SLC34A2 in counteracting innate immunity.

## MATERIALS AND METHODS

### Cell culture

HEK293T (ATCC-CRL-3216), U2OS (ATCC-HTB-96), VERO (ATCC-CCL-81), A549 (CRM-CCL-185), HFF (ATCC-SCRC-1041), and NIH3T3 (ATCC-CRL-1658) cells were cultured in Dulbecco’s Modified Eagle’s Medium (DMEM; Gibco) supplemented with 10% fetal bovine serum (FBS; LONSERA, Shanghai, China) and 1% penicillin-streptomycin (Gibco). OVCAR-3 (Cell Resource Center, CAMS/PUMC, Beijing, China) cells were cultured in RPMI 1640 supplemented with 20% FBS, 1% penicillin-streptomycin, and 0.01 mg/mL insulin (MedChemExpress). BCBL-1 cells were kindly provided by Dr. Ke Lan (Wuhan University) (53, 54). BCBL-1 and THP-1 (ATCC-TIB-202) cells were cultured in RPMI 1640 (Gibco) supplemented with 10% FBS and 1% penicillin-streptomycin. BCBL-1-Tet-RTA cells were generated by stably transducing BCBL-1 with a doxycycline-inducible RTA construct (55). For cell culture under phosphate-limited conditions, THP-1 cells were cultured in phosphate-free DMEM (Gibco) supplemented with 10% FBS and 1% penicillin-streptomycin. For cell culture under near-physiological phosphate conditions, THP-1 cells were cultured in DMEM (Gibco) supplemented with 10% FBS and 1% penicillin-streptomycin. Primary mouse lung fibroblasts (MLFs) were generated and cultured as previously described (56, 57). CD14^+^ peripheral blood mononuclear cells (PBMCs), sorted from healthy volunteers with informed consent from all participants using a negative selection method (Milestone Biotechnology, Shanghai, China), were cultured in RPMI-1640 medium supplemented with 10% fetal bovine serum (FBS), and 1% penicillin-streptomycin.

### Viruses

Herpes simplex virus type 1 (HSV-1), HSV-1-GFP, and VSV were propagated using VERO cells (56, 58, 59). HCMV was kindly provided by Drs. Hong-Bing Shu and Qing Yang (Wuhan University) and propagated using HFF cells. MCMV was kindly provided by Drs. Min-Hua Luo and Bo Yang (Wuhan Institute of Virology) and propagated using NIH3T3 cells. MHV68 was kindly provided by Dr. Hongyu Deng (Institute of Biophysics, Chinese Academy of Sciences) and propagated using NIH3T3 cells (60, 61). SeV was purchased from Charles River Laboratories (62). PRV was kindly provided by Dr. Zhengfei Liu (Huazhong Agricultural University) and propagated using VERO cells. VACV was kindly provided by Drs. Yun Wang and Yang Qiu (Wuhan Institute of Virology) and propagated using VERO cells. HAdV was kindly provided by Dr. Qiwei Zhang (Jinan University) and propagated using VERO cells. Virus titer was determined by standard plaque assay by using VERO, HFF, or NIH3T3 cells.

### Constructs and reagents

For transient expression in mammalian cells, *DUX4* and its mutants (N144A, R148A, and N69A) were constructed into pEF-EF1α-FLAG-N vector. For stable cell line generation, *DUX4*, *SLC34A2*, *ZSCAN4*, *CPHXL*, *FRG2*, and *MBD3L2* were amplified from cDNA and subcloned into pLVX-TetOne (Takara) with a FLAG tag. For the rescue experiments, *DUX4* and its mutant (N144A) with synonymous mutations at the shRNA (#1) targeting site were constructed into pLVX-TetOne. Mouse *Dux* was genetically synthesized and cloned into pLVX-TetOne with a FLAG tag. *IRF3* was amplified from pRK-FLAG-IRF3 (kindly provided by Dr. Hong-Bing Shu, Wuhan University) (63) and subcloned into pGEX-6P-1 (GE Healthcare). *TBK1* was amplified from pcDNA3-FLAG-TBK1 (kindly provided by Dr. Pinghui Feng, University of Southern California), and subcloned into pEF-FLAG.

To construct the pGL4-DUX4-luciferase-reporter plasmid, the promoter region of *ZSCAN4* containing the DUX4 binding site was amplified from genomic DNA and sub-cloned into pGL4 (Promega). sgRNAs targeting *FRG2*, *CPHXL*, *SLC34A2*, *MBD3L2/3/4/5*, *ZSCAN4*, *DSPP*, *TRIM49* were constructed into Lenti-CRISPRv2 (52961, Addgene). shRNAs targeting *DUX4* and *TRIM43* were cloned into pLKO.1-puro (8453, Addgene). siRNAs targeting *DUX4* and *SLC34A2* were synthesized by GeneCreate (Wuhan, China). The sequences for sgRNA, shRNA, and siRNA used in this study were listed in Table S1.

The chemical reagents used in this study include HT-DNA (Sigma-Aldrich), doxycycline (Sigma-Aldrich), poly I:C, puromycin, hygromycin B, and blasticidin (InvivoGen), diABZI (Selleck), NaPi2b-IN-2 and 5-Azacytidine (MedChemExpress), Lipofectamine 3000 (Thermo).

### Reporter assay

Luciferase reporter assays were performed using a dual luciferase assay system (Promega) (55). HEK293T cells in 24-well plates were transfected with a plasmid mixture containing 50 ng of the plasmid expressing IFN-β firefly luciferase reporter or DUX4 firefly luciferase reporter, and 20 ng of the plasmid expressing TK-Renilla luciferase reporter. At 24 h post-transfection, cells were harvested, and cell lysates were prepared. Luciferase activities were measured according to the manufacturer’s instructions (Promega).

### RNA extraction and RT-qPCR

Human THP-1 or OVCAR-3 cells were infected with HSV-1 (MOI = 1 or 5), VSV (MOI = 5), or SeV (20 HAU/mL), transfected with HT-DNA (1 µg/mL) or poly I:C (1 µg/mL), or stimulated with diABZI (2.5 µM). Mouse MLFs were infected with HSV-1 (MOI = 1 or 5), VSV (MOI = 5), PRV (MOI = 1), MCMV (MOI = 1), or MHV68 (MOI = 1). BCBL-1-Tet-RTA cells were treated with Dox (1 µg/mL) and sodium butyrate (0.2 mM) for 24 h. A549 cells were infected with HAdV (MOI = 1) or MCMV (MOI = 1). HEK293T cells were infected with VACV (MOI = 2) or MHV68 (MOI = 1). U2OS cells were infected with PRV (MOI = 1) for 24 h. HFF cells were infected with HSV-1 (MOI = 1) or HCMV (MOI = 0.5). Monocytes were transfected with 100 nM siRNA and infected with HSV-1 (MOI = 1) after 16 h post-transfection. Then, the cells were washed with ice-cold phosphate-buffered saline (PBS) at the indicated time points, and total RNA was extracted using TRIzol reagent (Takara). The extracted RNA was treated with DNase I (New England Biolabs) to remove genomic DNA contamination. One microgram of total RNA was used for reverse transcription with HiScript II Reverse Transcriptase (Vazyme, Nanjing, China) according to the manufacturer’s instructions. qPCR was performed with the SYBR Green qPCR Master Mix (Bimake, Shanghai, China) using the Bio-Rad CFX Connect Real-Time PCR Detection System. The expression level of the target genes was normalized to housekeeping genes (*ACTB* for human genes and *Actb* for mouse genes). The primers (50, 64) used for qPCR were provided in Table S2.

### RNA-seq

THP-1 cells (2  ×  10^6^, three replicates per sample) were mock-infected or infected with HSV-1 (MOI = 1) for 12 h, and total RNA was isolated with the TRIzol reagent (Takara) according to the manufacturer’s instructions. Oligo(dT)-coupled magnetic beads were used to purify mRNA, and then a sequencing library was constructed. RNA sequencing was performed with the BGISEQ500 platform (BGI). The raw sequencing data were processed using SOAPnuke (v.1.5.2) to remove low-quality reads, and the clean reads were mapped to the reference genome using HISAT2 (v.2.0.4). Bowtie2 (v.2.2.5) was employed to align the clean reads to the reference coding gene set, and RSEM (v.1.2.12) was used to quantify gene expression levels. Differential gene expression analysis was conducted using DESeq2 (v.1.4.5), with a log2 fold change ≥2 and *P*-value ≤ 0.01 used to define differentially expressed genes (DEGs). A heatmap was created with pheatmap (v.1.0.8). The RNA sequencing data have been uploaded to the GEO database (accession number: GSE279339).

### Measurement of intracellular phosphate

Intracellular phosphate was quantified with a Malachite Green Phosphate Detection Kit (Beyotime, Shanghai, China) according to the manufacturer’s guidelines. Cells subjected to the indicated treatments were harvested and lysed in lysis buffer (10 mM HEPES pH 7.5, 1% TritonX-100, supplemented with protease inhibitors), and the cell lysates were centrifuged at 12,000 rpm for 10 min. The supernatants or a series of phosphate standards were diluted to a final volume of 100 µL with water in a 96-well microtiter plate, followed by the addition of malachite green chromogen (35 µL). The absorbance at 630 nm was measured with a microplate reader after 30 min. If any samples fell outside the linear range of the assay, the samples were rediluted and reanalyzed. The intracellular phosphate levels were calculated by dividing the calculated phosphate concentrations by the corresponding protein amounts (determined by BCA assay).

### *In vitro* kinase assay

GST-IRF3 was purified from *Escherichia coli* with glutathione agarose (Smart-lifesciences, Changzhou, China) and visualized by Coomassie Brilliant Blue staining, while FLAG-TBK1 was purified from HEK293T cells with FLAG agarose (Dia-An Biotechnology, Wuhan, China) as previously described (65). For the *in vitro* kinase assay, 1 µg of GST-IRF3 was incubated with 100 ng of FLAG-TBK1 in kinase assay buffer (20 mM HEPES pH 7.5, 100 mM NaCl, 10 mM MgCl_2_, 2 mM DTT, 300 µM ATP) for 2 h at room temperature. The samples were then resolved by SDS-PAGE and analyzed by immunoblotting.

### Immunoprecipitation and immunoblotting

For immunoprecipitation, THP-1 cells were mock-transfected or transfected with HT-DNA, and whole cell lysates (WCLs) were prepared at 3 h post-transfection. The WCLs were incubated with 1 µg of STING antibody plus 10 µL of protein G-agarose (GE Healthcare) at 4°C for 4 h. The agarose beads were then washed three times with lysis buffer (50 mM Tris-HCl, pH 7.5, 150 mM NaCl, 1% NP-40, 1 mM EDTA, supplemented with protease inhibitors), and boiled with 1× SDS sample buffer at 95°C for 10 min. Precipitated proteins were resolved by SDS-PAGE and analyzed by immunoblotting.

To detect endogenous DUX4 protein, THP-1 cells subjected to the indicated treatments were incubated with sucrose lysis buffer (10 mM HEPES pH 7.9, 340 mM sucrose, 3 mM CaCl_2_, 2 mM magnesium acetate, 0.1 mM EDTA, 0.5% NP-40, supplemented with protease inhibitors) for 10 min on ice. The samples were centrifuged at 3,500×*g* for 5 min at 4°C. The supernatants were collected as the cytoplasmic fractions. The remaining pellets were washed twice with sucrose lysis buffer without NP-40 and then released by boiling in 1× SDS sample buffer for 20 min. Proteins were separated by SDS-PAGE and analyzed by immunoblotting.

A list of antibodies used in this study was provided in Table S3.

### cGAMP measurement

THP-1 stable cell lines were induced with doxycycline (0.5 µg/mL) for 24 h, and then transfected with HT-DNA (1 µg/mL). At 3 h post-transfection, the cells were pelleted and resuspended in lysis buffer (50 mM Tris-HCl pH 7.5, 150 mM NaCl, 1% (v/v) NP-40, 1 mM EDTA, supplemented with protease inhibitors) for 30 min on ice. The cell lysates were centrifuged at 15,000×*g* for 10 min at 4°C. The supernatants were collected and used for cGAMP ELISA assay (Cayman) according to the manufacturer’s instructions. Protein concentration in the supernatants was measured using a BCA protein assay kit and used to normalize cGAMP concentration.

### Flow cytometry

For flow cytometry staining of primary human monocytes, cells were blocked with Fc block (BioLegend) for 10 min at 4°C, followed by incubation with fluorescently conjugated antibodies CD45-PE (BioLegend) and CD14-BV421 (BioLegend) for 30 min at 4°C. The stained cells were analyzed using a BD FACSCelesta Cell Analyzer (BD Biosciences). All flow cytometry data were analyzed with FlowJo v10 (BD Biosciences).

### Statistical analysis

Data represent the means of at least three independent experiments, and error bars denote standard deviations (SD). A two-tailed Student’s t test or analysis of variance was used for statistical analysis. GraphPad Prism 8 was used for statistical analysis. Significant differences are represented by *P*-value (*P < 0.05, **P < 0.01, ***P < 0.001, ****P < 0.0001, N.S., not statistically significant).

## SUPPLEMENTAL MATERIAL

Fig. S1 mbio.02554-25-s0001.tif HSV-1 infection induces DUX4.

Fig. S2 mbio.02554-25-s0002.tif Species-specific induction of DUX4 by herpesviruses.

Fig. S3 mbio.02554-25-s0003.tif DUX4 suppresses antiviral innate immunity dependent on its transcriptional activity.

Fig. S4 mbio.02554-25-s0004.tif SLC34A2 induced by DUX4 is required to suppress antiviral innate immunity.

Fig. S5 mbio.02554-25-s0005.tif SLC34A2 modulates intracellular inorganic phosphate levels to restrict antiviral innate immunity.

Fig. S6 mbio.02554-25-s0006.tif Targeted inhibition of SLC34A2 enhances antiviral innate immune responses.

Supplemental text mbio.02554-25-s0007.docx Supplemental figure legends.

Table S1 mbio.02554-25-s0008.xlsx sgRNA, shRNA, and siRNA.

Table S2 mbio.02554-25-s0009.xlsx Primer information.

Table S3 mbio.02554-25-s0010.xlsx Antibody information.

ASM does not own the copyrights to Supplemental Material that may be linked to, or accessed through, an article. The authors have granted ASM a non-exclusive, world-wide license to publish the Supplemental Material files. Please contact the corresponding author directly for reuse.
