# Supplementary figures and images for: Human-specific activation of the DUX4-SLC34A2 axis by herpesviruses suppresses antiviral innate immunity
Source: mBio. 2025 Nov 10;16(12):e02554-25. doi: 10.1128/mbio.02554-25 (PMC12691587; doi:10.1128/mbio.02554-25)

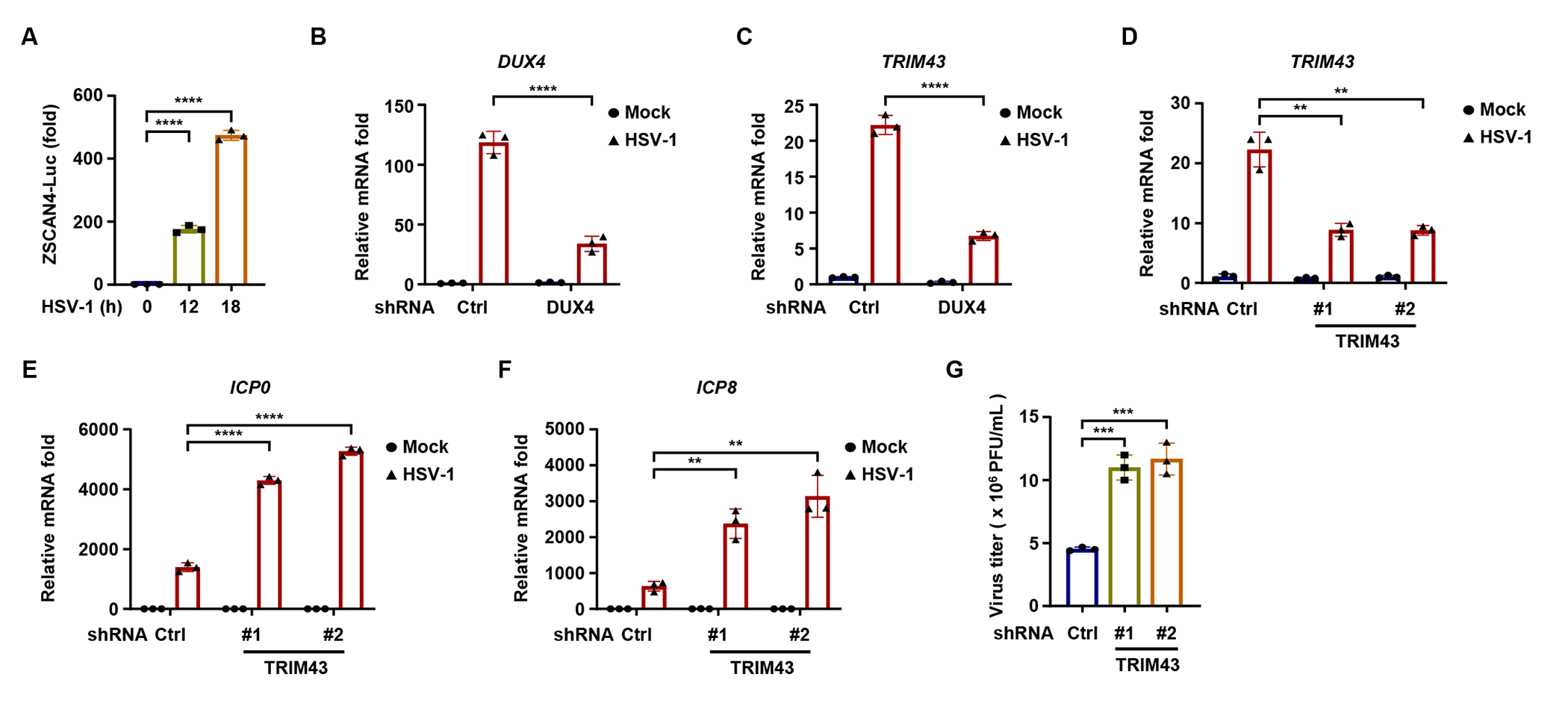

Supplement: Fig. S1 — HSV-1 infection induces DUX4. [file mbio.02554-25-s0001.tif]

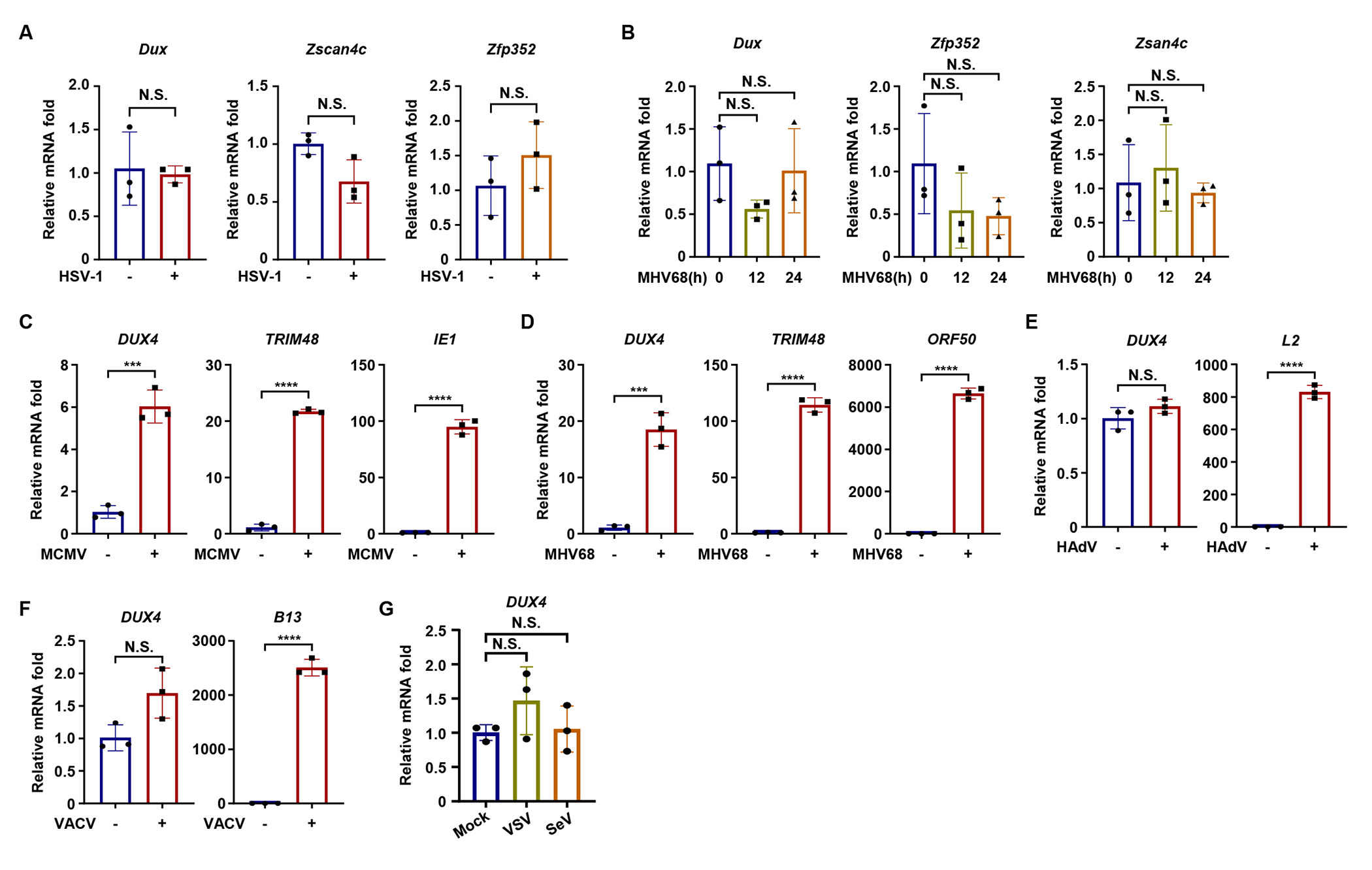

Supplement: Fig. S2 — Species-specific induction of DUX4 by herpesviruses. [file mbio.02554-25-s0002.tif]

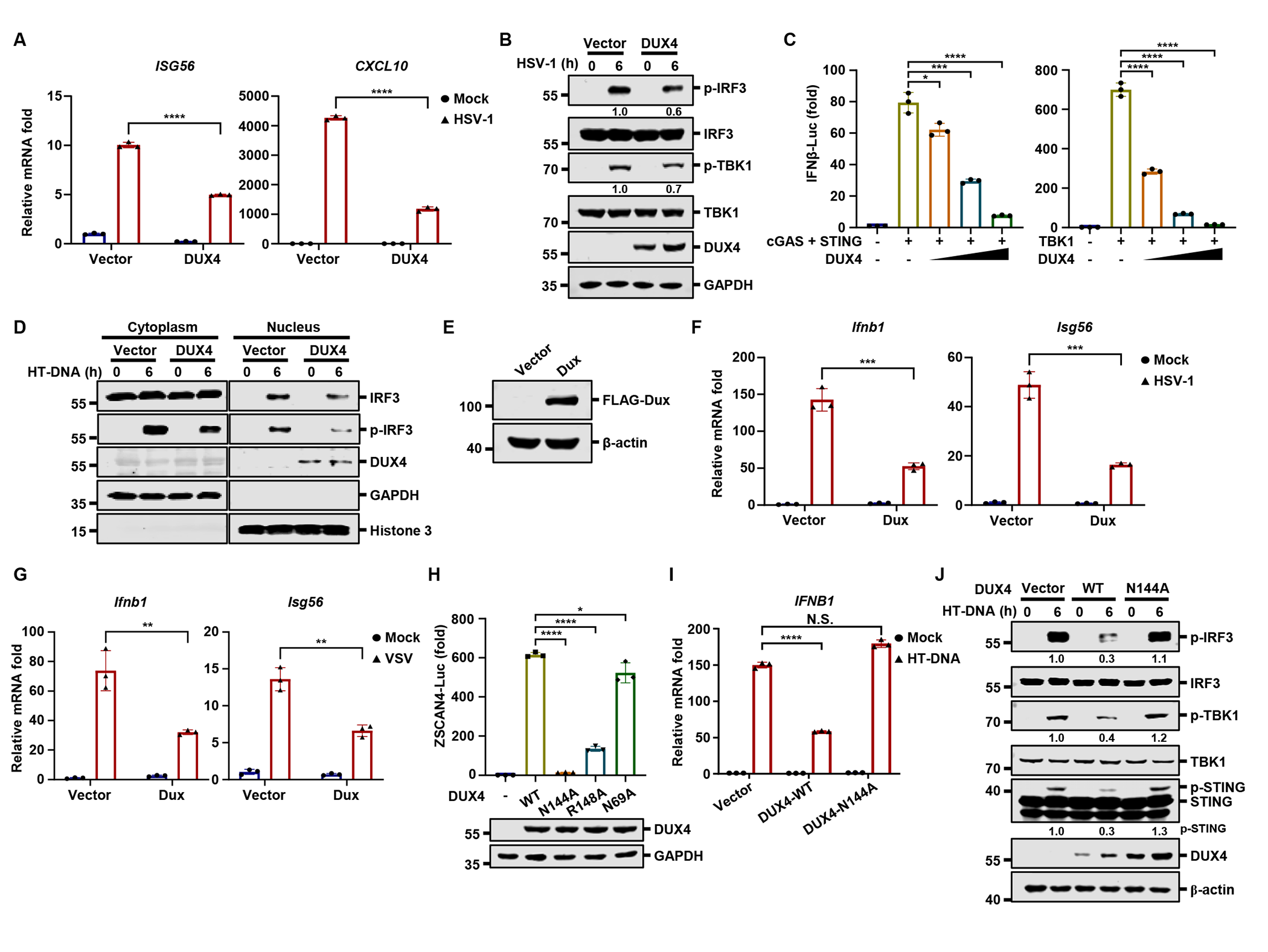

Supplement: Fig. S3 — DUX4 suppresses antiviral innate immunity dependent on its transcriptional activity. [file mbio.02554-25-s0003.tif]

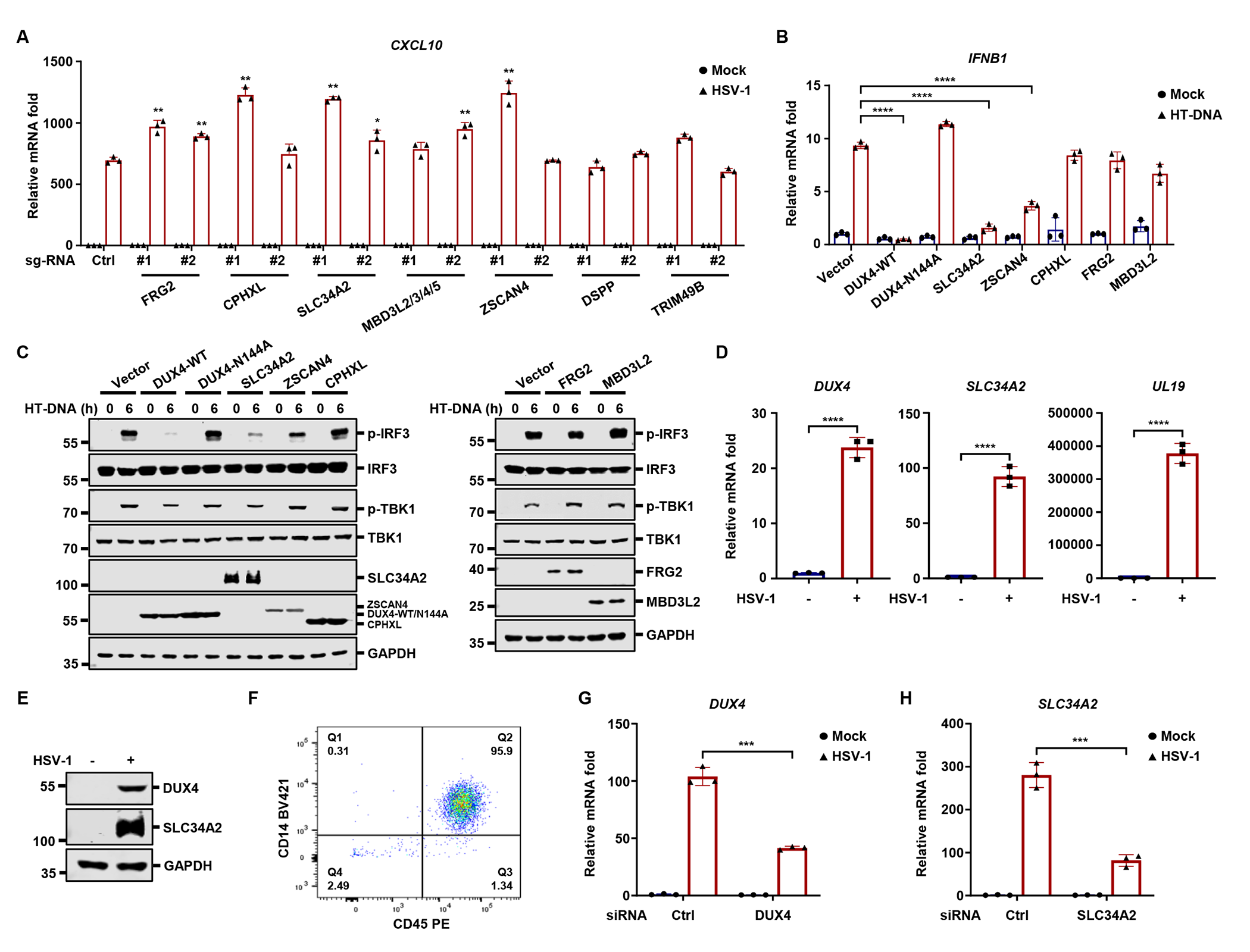

Supplement: Fig. S4 — SLC34A2 induced by DUX4 is required to suppress antiviral innate immunity. [file mbio.02554-25-s0004.tif]

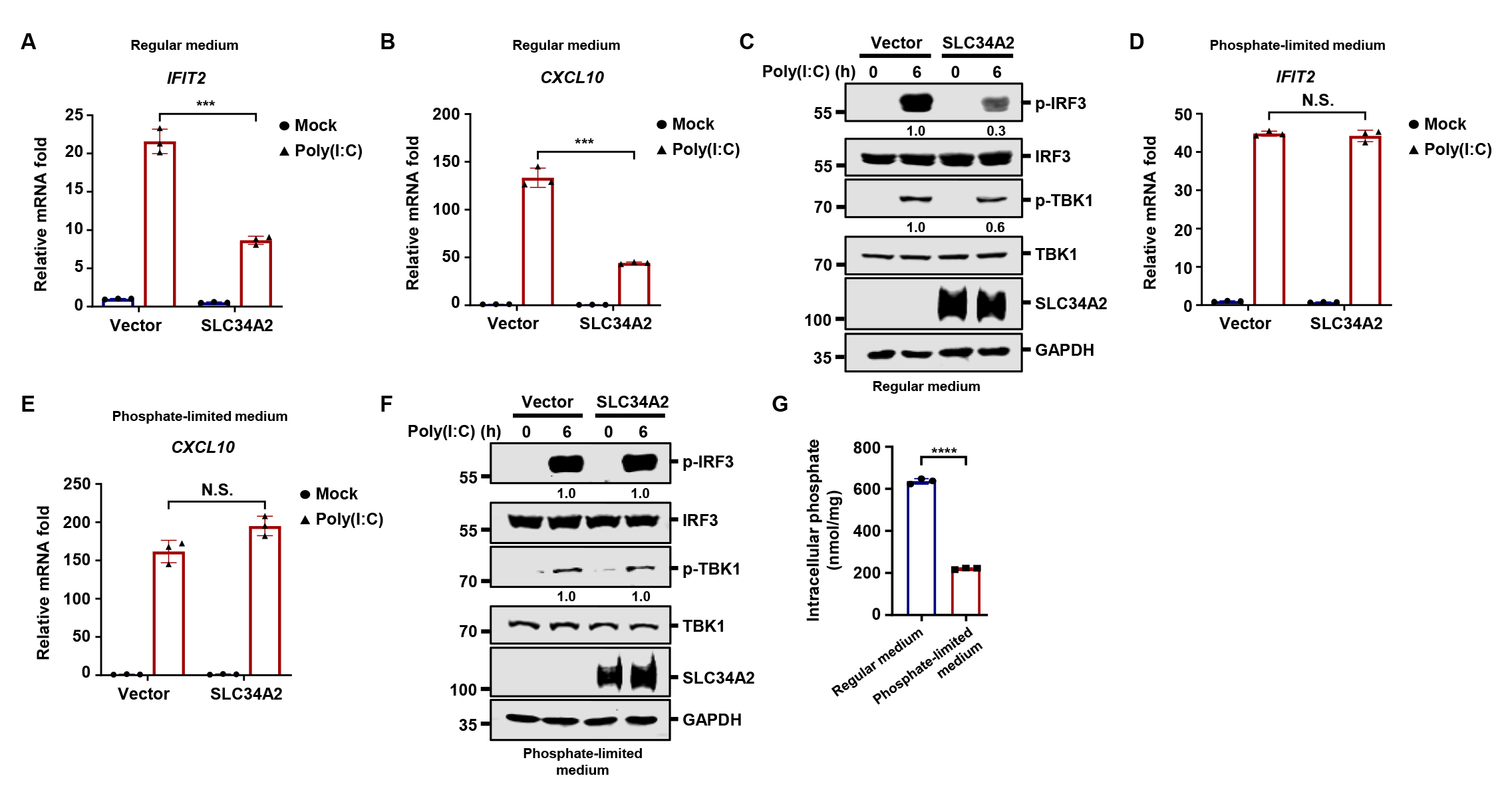

Supplement: Fig. S5 — SLC34A2 modulates intracellular inorganic phosphate levels to restrict antiviral innate immunity. [file mbio.02554-25-s0005.tif]

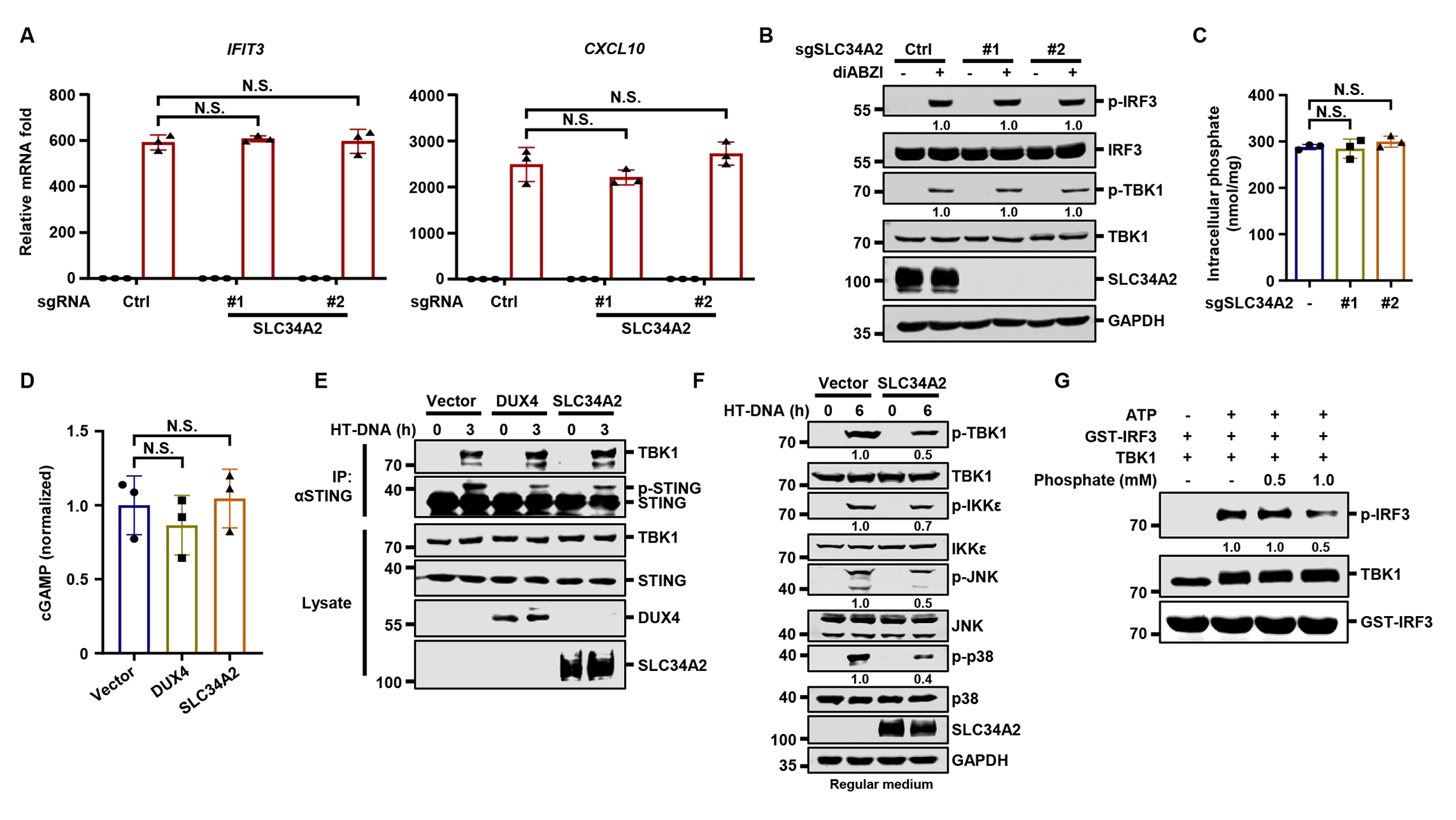

Supplement: Fig. S6 — Targeted inhibition of SLC34A2 enhances antiviral innate immune responses. [file mbio.02554-25-s0006.tif]
