# Supplementary material for: Human-specific activation of the DUX4-SLC34A2 axis by herpesviruses suppresses antiviral innate immunity
Source: mBio. 2025 Nov 10;16(12):e02554-25. doi: 10.1128/mbio.02554-25 (PMC12691587; doi:10.1128/mbio.02554-25)
Supplement: Supplemental text — Supplemental figure legends. [file mbio.02554-25-s0007.docx]

**Figure S1** **HSV-1 infection induces DUX4**

(A) HEK293T cells were transfected with a luciferase reporter plasmid driven by *ZSCAN4* promoter, and then the cells were infected with HSV-1 (MOI=1) at 18 h post-transfection. Luciferase activities were determined at the indicated time points post-infection.

(B-C) HFF cells transduced with control shRNA (Ctrl) or shRNA targeting *DUX4* were mock-infected or infected with HSV-1 (MOI=1). The expression of the indicated genes was quantified at 12 h post-infection.

(D-G) HFF cells transduced with control shRNA (Ctrl) or shRNA targeting *TRIM43* were mock-infected or infected with HSV-1 (MOI=1). The expression of the indicated genes was quantified at 12 h post-infection (D-F). Viral titers in the supernatants were quantified at 24 h post-infection (G).

**Figure S2 Species-specific induction of DUX4 by herpesviruses**

(A) MLF cells were infected with HSV-1 (MOI=1), and the expression of the indicated genes was quantified at 12 h post-infection.

(B) MLF cells were infected with MHV-68 (MOI=1), followed by quantification of the indicated genes at the indicated time points post-infection.

(C and D) A549 cells were infected with MCMV (MOI=1) for 3 days (C), and HEK293T cells were infected with MHV68 (MOI=1) for 24 h (D). The expression of the indicated genes was quantified by RT-qPCR.

(E and F) A549 cells were infected with HAdV (MOI=1) for 18 h (E), and HEK293T cells were infected with VACV (MOI=2) for 12 h (F). The expression of the indicated genes was quantified by RT-qPCR.

(G) THP-1 cells were infected with VSV (MOI=5) or SeV (20 HAU/ml), and the expression of *DUX4* was quantified at 10 h post-infection.

**Figure S3 DUX4 suppresses antiviral innate immunity dependent on its transcriptional activity**

(A and B) THP-1 cells stably expressing doxycycline-inducible vector control or *DUX4* were treated with doxycycline for 24 h, and then infected with HSV-1 (MOI=5). The expression of *ISG56* and *CXCL10* was quantified at 6 h post-infection (A), and WCLs were analyzed by immunoblotting at the indicated time points post-infection (B).

(C) HEK293T cells were transfected with an IFN-β promoter reporter plasmid mixture, and different amounts of DUX4 (0, 0.1, 0.2 or 0.5 μg). Luciferase activities were determined at 24 h post-transfection.

(D) THP-1 cells stably expressing doxycycline-inducible vector control or *DUX4* were treated with doxycycline for 24 h, and then transfected with HT-DNA (1 μg/ml). Nuclear and cytoplasmic fractions were isolated and analyzed by immunoblotting.

(E) MLF cells stably expressing doxycycline-inducible vector control or *Dux* were treated with doxycycline for 24 h, the WCLs were analyzed by immunoblotting.

(F) MLF cells stably expressing doxycycline-inducible vector control or *Dux* were treated with doxycycline for 24 h, and then infected with HSV-1 (MOI=5). The expression of *Ifnb1* and *Isg56* was quantified at 6 h post-infection.

(G) MLF cells stably expressing doxycycline-inducible vector control or *Dux* were treated with doxycycline for 24 h, and then infected with VSV (MOI=5). The expression of *Ifnb1* and *Isg56* was quantified at 6 h post-infection.

(H) HEK293T cells were transfected with a *ZSCAN4* promoter luciferase reporter plasmid and WT DUX4 or the indicated mutants. Luciferase activities were determined at 24 h post-transfection.

(I and J) THP-1 stably expressing doxycycline-inducible vector control or the indicated genes were treated with doxycycline (500 ng/ml) for 24 h, and then transfected with HT-DNA (1 μg/ml). The expression of *IFNB1* was analyzed by RT-qPCR (I), and WCLs were analyzed by immunoblotting at 6 h post-transfection (J).

**Figure S4 SLC34A2 induced by DUX4 is required to suppress antiviral innate immunity**

(A) THP-1 cells transduced with control sgRNA or sgRNA targeting the indicated genes were infected with HSV-1 (MOI=5), and the expression of *CXCL10* was analyzed by RT-qPCR at 8 h post-infection.

(B and C) THP-1 cells stably expressing doxycycline-inducible vector control or the indicated genes were treated with doxycycline for 24 h, and then transfected with HT-DNA (1 μg/ml). The expression of *IFNB1* was analyzed by RT-qPCR (B), and WCLs were analyzed by immunoblotting at 6 h post-transfection (C).

(D and E) SH-SY5Y cells were infected with HSV-1 (MOI=1), and the expression of indicated genes was quantified at 12 h post-infection (D). WCLs were analyzed by immunoblotting at 12 h post-infection (E).

(F) Flow cytometry analysis of primary human monocytes stained with CD45-PE and CD14-BV421 antibodies.

(G and H) Primary human monocytes transfected with control siRNA or siRNA targeting *DUX4* or *SLC34A2* were infected with HSV-1 (MOI=1) at 16 h post-transfection. The expression of the indicated genes was quantified at 12 h post-infection.

**Figure S5 SLC34A2 modulates intracellular inorganic phosphate levels to restrict antiviral innate immunity**

(A-C) THP-1 cells stably expressing doxycycline-inducible vector control or *SLC34A2* were induced with doxycycline for 24 h under high phosphate culture condition (50 mg/dL), and then were transfected with poly I:C (1 μg/ml). The expression of *IFIT2* and *CXCL10* was quantified by RT-qPCR at 6 h post-transfection (A and B), and WCLs were analyzed by immunoblotting (C).

(D-F) THP-1 cells stably expressing doxycycline-inducible vector control or *SLC34A2* were induced with doxycycline for 24 h under phosphate-limited culture condition, and then were transfected with poly I:C (1 μg/ml). The expression of *IFIT2* and *CXCL10* was quantified by RT-qPCR at 6 h post-transfection (D and E), and WCLs were analyzed by immunoblotting (F).

(G) THP-1 cells were cultured in either standard medium or phosphate-limited medium, followed by quantification of intracellular phosphate concentration.

**Figure S6 Targeted inhibition of SLC34A2 enhances antiviral innate immune responses**

(A-B) OVCAR-3 cells transduced with control sgRNA or sgRNA targeting *SLC34A2* were cultured under phosphate-limited culture conditions and stimulated with diABZI (2.5 μM). The expression of the indicated genes was quantified by RT-qPCR (A), and WCLs were analyzed by immunoblotting at 6 h post-stimulation (B).

(C) OVCAR-3 cells transduced with control sgRNA or sgRNA targeting *SLC34A2* were cultured under phosphate-limited conditions, followed by quantification of intracellular phosphate concentration.

(D and E) THP-1 stably expressing doxycycline-inducible vector control, *DUX4,* or *SLC34A2* were induced with doxycycline for 24 h, and then transfected with HT-DNA for 3 h. Intracellular cGAMP was quantified by ELISA (D). WCLs were collected and co-immunoprecipitation was performed with STING antibody. Precipitated proteins and WCLs were analyzed by immunoblotting (E).

(F) THP-1 stably expressing doxycycline-inducible vector control or *SLC34A2* were induced with doxycycline for 24 h under high phosphate culture condition, and then were transfected with HT-DNA (1 μg/ml). The WCLs were analyzed by immunoblotting at 6 h post-transfection.

(G) *In vitro* kinase assay was performed using purified FLAG-TBK1 and GST-IRF3 supplemented with or without phosphate.
